# Variable Presentation of the CYBB Mutation in One Family, Approach to Management, and a Review of the Literature

**DOI:** 10.1155/2020/2546190

**Published:** 2020-02-06

**Authors:** Tatyana Gavrilova, Ari Zelig, Diana H. Lee

**Affiliations:** ^1^Division of Allergy and Immunology, Montefiore Medical Center, Albert Einstein College of Medicine, 1525 Blondell Avenue, Bronx, NY 10461, USA; ^2^Department of Dermatology, Montefiore Medical Center, Albert Einstein College of Medicine, 3415 Bainbridge Avenue, Bronx, NY 10467, USA

## Abstract

Chronic granulomatous disease (CGD) is a primary immunodeficiency disorder marked by abnormal phagocytic function. CGD affects primarily neutrophils and manifests as an early predisposition to severe life-threatening infections. Additionally, patients with CGD are predisposed to unique autoimmune manifestations. While generally spared from infectious complications, heterozygous carriers of the abnormal genes implicated in CGD pathogenesis can still present with autoimmune disorders. A mutation in the CYBB gene is the only X-linked variant of this disease. This article describes a family with the CYBB mutation, its heterogenous presentation, and reviews the literature discussing disease management.

## 1. Introduction

Chronic granulomatous disease (CGD) is a primary immunodeficiency disorder that results in impaired phagocytic function. X-linked inheritance pattern is most common, while others are autosomal recessive. However, in countries with frequent consanguineous marriage, autosomal recessive inheritance is more commonly reported [1, 2]. Patients with CGD are predisposed to invasive, life-threatening infections as well as diseases marked by immune dysregulation, such as autoimmune disorders. Interestingly, heterozygous carriers of these mutations, while usually spared from infectious complications, carry the same risk for autoimmune disease. This report describes siblings with the pathogenic CYBB mutation as well as reviews the literature of this disease and management of these patients.

## 2. Case Presentation

### 2.1. Female CYBB Carrier

GN was a 5-month-old female patient at the time of her initial presentation to our clinic. She was a product of a nonconsanguineous union and born at 44 weeks via vaginal delivery. She had two older deceased male siblings, and it is unknown if these two brothers shared the same father. The oldest brother passed away at the age of 3 months of a suspected unspecified infection. Two weeks prior to the patient's initial presentation to us, her second brother passed away from sepsis at the age of five years. This brother was confirmed to have X-linked CGD at an outside institution where he was unfortunately lost to follow-up. The mother said that she was tested at the outside institution as well and was found to be a carrier for the same pathogenic mutation. She denied a personal history of significant or life-threatening infections or history of autoimmunity.

At the age of 2 months, GN started to develop recurrent oral ulcers. She also had a chronic waxing and waning rash on her face and extremities. The rash would flare after receiving vaccines and during bouts of viral respiratory tract infections. The patient had one episode of coxsackie virus and no history of bacterial or fungal infections. She had recurrent oral mucosal erosions, a violaceous periorbital rash, and malar edematous erythematous patches and plaques. Serial infectious workup of the oral lesions was negative. A skin biopsy showed an interface dermatitis with a primarily lymphocytic infiltrate at the dermoepidermal junction with perivascular involvement. Most of the cells in the infiltrate expressed CD68 and myeloperoxidase, features of immature myeloid cells. This was a primarily T-cell infiltrate as most cells were CD3+, and there were few CD20 + cells. The skin biopsy was negative for bacteria, acid fast bacilli, and fungi. Her oral and cutaneous lesions responded to fluocinonide 0.05% gel (mouth) and fluocinonide ointment to affected areas of skin. Interestingly, GN's recently deceased brother had a similar rash on his face and his skin biopsy identified interface dermatitis with a mild granulomatous infiltrate.

Genetic testing confirmed the patient to be a heterozygous carrier of the pathogenic CYBB p.Ile248AsnfsX36 variant. She also had a CYBA mutation p.Glu135Lys of uncertain significance. Her neutrophil oxidative burst was initially 46% but normalized to 99% when rechecked.

GN also had intermittent neutropenia with a nadir of 600 K/*μ*L. Anti-neutrophil IgM was weakly positive. Over time, the neutrophil count trended up and stabilized at 1.4 K–2.2 K/*μ*L. A bone marrow biopsy was considered but declined by the parent. She did not have hypocomplementemia, and aside for a mildly positive ANA (40 units, homogenous pattern) and a proteinase 3 (PR3) antibody positive at 1.2 AI (0–0.9), the remainder of her rheumatologic workup was negative including ds-DNA, aldolase, SSA/Ro, and SSB/La.

### 2.2. Male CYBB CGD

LN, GN's brother, presented for evaluation two weeks after birth for genetic testing due to the significant family history. The DHR oxidative burst was 6%, and he tested positive for the same pathogenic CYBB variant as his sister. The patient was started on prophylactic antimicrobial agents with trimethoprim/sulfamethoxazole and voriconazole, which was then changed to itraconazole, as well as interferon gamma-1b. The serum level of the antifungal prophylactic agent continued to be subtherapeutic despite loading and weight-appropriate dosing, likely secondary to medication noncompliance. Despite the early referral for HSCT and counseling, the parent declined to pursue this curative option before 12 months of age. LN's maternal grandmother requested to have genetic testing be done for her as well in the hopes that she can serve as his bone marrow donor. Interestingly, she was found to not have the CYBB mutation implying that the patient's mother had a de novo pathogenic CYBB mutation.

The patient had a complicated infectious history with several bouts of pubic cutaneous lesions that grew *Klebsiella pneumonia*, MDR *Enterobacter asburiae,* and *Serratia marcescens*. He had a bout of unilateral bacterial conjunctivitis that was positive for *Serratia marcescens* and *Neisseria sicca/subflava*. LN also experienced recurrent *Clostridium difficile* colitis. His pretransplant course was further complicated by ESBL *Klebsiella pneumonia*, *Enterobacter cloacae* sepsis, and *Klebsiella* bacteremia. He was started on hydroxyurea and azathioprine in preparation for transplant, but this regimen was discontinued due to *Candida Parapsilosis* bacteremia. He commenced cytoreduction therapy with busulfan, melphalan, fludarabine, and ATG and received a T-cell depleted unrelated 9/10 matched HSCT at 19 months of age. He obtained 100% donor chimerism with a normalized oxidative burst and discharged with outpatient follow-up.

## 3. Discussion

CGD is a primary immunodeficiency disorder of the NADPH oxidase complex that results in a phagocytic functional defect secondary to the impairment of reactive oxygen species (ROS) production. The impairment of neutrophils and monocytes results in recurrent severe life-threatening infections. The immune dysregulation, however, also results in autoimmunity which carries its own significant risk of morbidity [3]. The overall incidence of CGD in the US is approximately 1/200,000 live births [4].

The NADPH oxidase complex is composed of the cell membrane-bound glycoprotein gp91^phox^ (CYBB gene) and nonglycosylated protein p22^phox^ (CYBA), as well as p47^phox^ (NCF1), p67^phox^ (NCF2), and p40^phox^ (NCF4), which are cytosolic proteins. Mutations in any of these components result in defective ROS production and clinical CGD manifestations. A mutation in the X-linked CYBB is responsible for approximately 65% of the cases with CGD. CYBA and NCF1 mutations account for 20% of CGD occurrence, NCF2 and CYBA mutations result in 5% of cases, and NCF4 being rarest with only one reported case [3, 5].

Patients with X-linked CGD generally have more severe disease due to the lower superoxide production than the autosomal recessive phenotypes. Most cases of CGD present in early childhood and typically as severe invasive infections. Catalase positive bacteria and fungi are the pathognomonic agents of these infections. Aspergillus is the most commonly isolated pathogen, while *Burkholderia* infection is associated with the greatest severity. *S. aureus*, *Nocardia*, and *Serratia* are also among the common pathogens associated with CGD [6]. Bacille Calmette–Guerin (BCG) and *Mycobacterium tuberculosis* are pathogens identified in developing countries [3].

Cutaneous manifestations of CGD include staphylococcal infections around the ear and nose during infancy, occasionally accompanied by lymphadenopathy. Patients can present with ecthyma gangrenosum as neonates. Cutaneous abscesses occur often, usually with *S. aureus*, but also with *Serratia marcescens*, and can at times heal poorly. Less often, there can be noninfectious purulent inflammatory reactions at sites of trauma or lymph nodes. Sterile cutaneous granulomas are often nodular and necrotic [7].

Patients with CGD and heterozygous carriers are at an equal risk for inflammatory and autoimmune complications. Aphthous stomatitis and photosensitive lupus-like skin eruptions have been reported in these patients. The lupus-like rash can have acute or chronic features. Interestingly, a feature of the CGD-associated rash that differs from a typical discoid lupus erythematosus (DLE) is the absence of adherent scales and atrophic scarring in old lesions. Additionally, few of the reported cases tested positive on direct immunofluorescence (DIF) [8–10]. Overt SLE has been reported in CGD [4] as has been immune thrombocytopenia (ITP). Myasthenia gravis has been documented in the same patient registry but only in one patient [4]. Cases of antiphospholipid syndrome and recurrent pericardial effusion have also been cited, as have been juvenile idiopathic arthritis (JIA) and IgA nephropathy [11]. Additionally, patients with CGD can form inflammatory and possibly obstructive granulomas in any organ including the eyes, lungs, liver, and gastrointestinal and genitourinary tracts. Our heterozygous carrier had lupus-like cutaneous manifestations along with aphthous stomatitis. She also had isolated neutropenia, and to the authors' knowledge, this is the first report of this cytopenia to be associated with the CYBB carrier status.

Female X-linked carriers of CGD are a unique subgroup of patients. Lyonization results in variable degrees of gp91^phox^ function. Cutaneous symptoms, particularly a photosensitive lupus-like rash (most frequently DLE-like) and recurrent aphthous ulcers, are the most common autoimmune manifestations. Granulomatous cheilitis, Raynaud phenomenon, and folliculitis can also be seen [12, 13]. However, depending on the degree of X-chromosome inactivation, some may be predisposed to infections as well. While there is a correlation between neutrophil oxidative capacity lower than 20% of normal and increased infections, there is otherwise no observation of increased infections [3, 14].

Among the invasive infections seen in patients with CGD are pneumonia, lymphadenitis, liver abscesses, and osteomyelitis. Skin infections can be severe at times with purulent abscesses that can become chronic and associated with lymphadenopathy and poor healing. *Serratia* in particular has been associated with skin ulcers [15]. Ecthyma gangrenosum is a rare ulcerating infection in CGD. It is caused by *Pseudomonas aeruginosa* and has been reported as a presenting cutaneous symptom of CGD [16]. Cutaneous infections must be differentiated from sterile granulomatous lesions as this will impact management. This may be difficult as noninfectious granulomas can sometimes be severe and necrotic in appearance, making culture and biopsy most important in differentiating between these entities.

Management of CGD revolves around managing infections as well as autoimmune manifestations. Patients with CGD are started on prophylactic antimicrobial agents with trimethoprim-sulfamethoxazole (TMP-SMX) being the preferred antibacterial drug and itraconazole or voriconazole for antifungal prophylaxis. Dicloxacillin and ciprofloxacin are options for patients with contraindications to the use of TMP-SMX. Posaconazole is an alternative to itraconazole or voriconazole, but its pharmacokinetic properties generally favor administration along with a high-fat meal and avoidance of proton pump inhibitors (PPIs) [17].

Interferon-*γ* is another prophylactic agent that is utilized in the treatment of CGD patients. Although not without conflicting findings as to its long-term benefit, this subcutaneously injected medication is a generally well-tolerated treatment that has been found to reduce the frequency of serious infections, and it should be strongly considered for patients who experience infections despite prophylactic antimicrobial use [18].

Corticosteroids have been used along with antimicrobials in the treatment of severe inflammation associated with infections. Systemic steroids have been utilized in treating liver abscesses and pneumonia [19]. Venegas-Montoya et al. reported two cases of liver abscesses that were not amenable to surgical drainage and were treated with antimicrobials and systemic corticosteroids. The steroid doses varied at prednisone 0.6 mg–1 mg/kg/day to complete 40 days [20, 21].

Inflammatory and autoimmune complications are a byproduct of the immune dysregulation that is present in CGD, and those with the X-linked inheritance pattern are at the highest risk for these disorders. The gastrointestinal tract is one of the most commonly affected organs, and early onset inflammatory bowel disease should prompt an evaluation for a primary immunodeficiency disorder such as CGD. A unique histologic feature of these granulomatous lesions is golden-yellow granular pigment-laden macrophages [9].

Treatment of IBD in CGD is a fine balance between immunosuppression of the autoinflammatory disease and maintaining antimicrobial immunocompetence. Systemic corticosteroids and azathioprine have been reported to be used [22]. Anti-TNF*α* agents are associated with a significant risk of severe infections [23] and therefore generally avoided in these patients. Ustekinumab, anti-IL-12/IL-23 agent, has been used in CGD-associated colitis with symptomatic improvement, but its use in the reported case was discontinued due to an infection, with recurrence of colitis symptoms [24]. The use of anti-IL-1 anakinra demonstrated mixed findings in regards to treatment of CGD-associated colitis. de Luca et al. found that treatment with this agent resulted in clinical improvement. Hahn and colleagues, however, found that this medication had limited use in reducing long-term disease severity [25, 26].

### 3.1. HSCT

Allogeneic hematopoietic stem cell transplantation (HSCT) still remains the only curative option for CGD [27]. Prior infections can increase posttransplant complications and therefore adversely affect outcomes. Historically, the use of myeloablative therapy was not standard due to the concern for infections in these already immunocompromised patients [28]. However, reports of reduced intensity conditioning (RIC) for CGD patients reported a high rate of graft failure. Segar et al. reported a 27 CGD patient cohort, and 23 of those patients received a myeloablative busulfan-based regimen with donors being HLA-identical siblings. The successful outcomes of this patient cohort suggested that myeloablative conditioning followed by transplant is a feasible option for these patients. Martinez and colleagues [29] reported the outcomes of eleven children after matched sibling (4/11) and unrelated donor (MUD, 7/11) transplantation with the mean age of 3.8 years. 70% of these patients had intractable infections or steroid-dependent CGD at the time of transplantation. Martinez reported 100% survival of all patients and stable engraftment with full donor chimerism in 9 of 11 patients with a follow-up range of 1–9 years. The conditioning regimen used for MUD recipients was busulfan, cyclophosphamide, fludarabine, and alemtuzumab. Hoenig et al. report a case of a hemizygous CYBB male patient who underwent a haploidentical HSCT after myeloablative conditioning with successful engraftment [30]. Parta and colleagues [31] reported the first case of a successful haploidentical transplantation and stable neutrophil engraftment using posttransplant high-dose cyclophosphamide in a male patient with a CYBB mutation who also had refractory infectious pericarditis.

### 3.2. Gene Therapy

Animal and ex vivo studies have shown promise in the use of gene therapy to correct underlying CGD mutations. De Ravin and colleagues showed successful ex vivo targeted integration of the gp91phox gene into an adeno-associated virus 6 vector that resulted in an improved NADPH oxidase activity of neutrophils [32]. In vitro studies have been able to utilize exon replacement in induced pluripotent stem cells (iPSCs) to restore gp91^phox^ expression [33]. Clinical trials studying the use of gene-modified autologous hematopoietic cells for the treatment of CGD continue to be underway.
